# Comparative Analysis of Machine Learning and Deep Learning Algorithms for Assessing Agricultural Product Quality Using NIRS

**DOI:** 10.3390/s24165438

**Published:** 2024-08-22

**Authors:** Jiwen Ren, Yuming Xiong, Xinyu Chen, Yong Hao

**Affiliations:** 1 School of Mechatronics and Vehicle Engineering, East China Jiaotong University, Nanchang 330013, China; renjiwen@ecjtu.jx.cn (J.R.); 2022038080400014@ecjtu.edu.cn (Y.X.); 2 Optoelectronics Department of Changzhou Institute of Technology, Changzhou 213000, China; chenxiny@czu.cn

**Keywords:** Gramian angular difference field, convolutional neural networks, near-infrared spectroscopy, coordinate attention, robust model

## Abstract

The success of near-infrared spectroscopy (NIRS) analysis hinges on the precision and robustness of the calibration model. Shallow learning (SL) algorithms like partial least squares discriminant analysis (PLS-DA) often fall short in capturing the interrelationships between adjacent spectral variables, and the analysis results are easily affected by spectral noise, which dramatically limits the breadth and depth of applications of NIRS. Deep learning (DL) methods, with their capacity to discern intricate features from limited samples, have been progressively integrated into NIRS. In this paper, two discriminant analysis problems, including wheat kernels and Yali pears as examples, and several representative calibration models were used to research the robustness and effectiveness of the model. Additionally, this article proposed a near-infrared calibration model, which was based on the Gramian angular difference field method and coordinate attention convolutional neural networks (G-CACNNs). The research results show that, compared with SL, spectral preprocessing has a smaller impact on the analysis accuracy of consensus learning (CL) and DL, and the latter has the highest analysis accuracy in the modeling results using the original spectrum. The accuracy of G-CACNNs in two discrimination tasks was 98.48% and 99.39%. Finally, this research compared the performance of various models under noise to evaluate the robustness and noise resistance of the proposed method.

## 1. Introduction

Near-infrared spectroscopy (NIRS) analysis technology has been widely used in agriculture [1,2,3], food [4,5,6], medicine [7,8,9], and other fields due to its fast and non-destructive characteristics [10]. Generally, the response of NIRS is characterized by weak absorption and overlapping peaks. Therefore, it is necessary to use Principal Component Analysis (PCA), partial least squares (PLS), and other chemometric methods to reveal the complex relationship between multiple variables and targets [11]. Due to the susceptibility of NIRS to the environment, instrument status, sample physical state, etc., it is necessary to introduce spectral preprocessing such as denoising, smoothing, and standardization to suppress or eliminate the interference before analysis [12]. To eliminate the influence of interfering variables on the model’s accuracy and robustness, several methods, including Monte Carlo uninformative variable elimination (MC-UVE) [13], competitive adaptive reweighted sampling (CARS) [14], iterative variable subset optimization (IVSO) [15], etc., have been introduced to select useful modeling variables [16]. Several recent studies on the use of NIRS in agricultural product detection have demonstrated the effectiveness of multivariate calibration models. They include some new model optimization algorithms, such as the genetic algorithm (GA), the random frog leaping algorithm (RFLA), and particle swarm optimization (PSO), and some classic shallow learning (SL) models, such as PLS and support vector regression (SVR) [17,18,19]. In the above research, multiple preprocessing and data cleaning methods were integrated into the SL methods. These improvements improved to some extent the performance of the model, but the shortcomings of the SL algorithm, such as the lack of non-linear ability and the inability to capture the interrelationships between variables, have not been well addressed. It is necessary to promote the updating of NIRS analysis algorithms.

In spectral analysis, the existence of outliers may lead to the instability of the analysis model and an increase in prediction errors. To overcome the negative influence of outliers in the analysis model, the consensus strategy is introduced in the model construction. Since consensus learning (CL) uses the integration results of multiple-member models, the existence of outliers will only affect individual models and have relatively little influence on the final prediction. Chen et al. [20] explored the feasibility of adopting NIRS and CL to improve the diagnosis of colorectal cancer: multiple weak learners were integrated to construct diagnostic models, and a linear discriminant classifier (LDA) was used as the random subspace method (RSM) for the weak learners. Zhao et al. [21] analyzed the reflection spectra of ginseng, and the decision tree (DT)-based random forest (RF) machine learning method was successfully used to establish the model for identifying the growth year of ginseng.

In the field of spectral-combined deep learning (DL) algorithms, the most important benefit of DL is automatic feature engineering, which reduces the burden of variable selection and preprocessing. In addition, one-dimensional convolutional neural networks have poor generalization and low accuracy. Research on converting one-dimensional (1D) signals into two-dimensional (2D) images by different methods, including energy grayscale images [22], angle–amplitude images [23], binary images [24], etc., and then classifying and recognizing them based on DL technology is growing. Hao et al. [25] established the DL model based on the NIRS stacked encoding of the Yali pear, which realized the non-destructive online detection of Yali pear pests. Pu et al. [26] encoded the terahertz (THz) time domain spectra of Pericarpium Citri Reticulatae (PCR) into Graham Angle Field (GAF) images and Markov Transition Field (MTF) images.

DL methods have exhibited great potential in the qualitative and quantitative analysis of NIRS due to their strong feature extraction ability and accurate analysis results. In this paper, to fully understand the application considerations and operational guidelines of DL methods in the construction of NIRS analysis models, two classification tasks of wheat kernels and Yali pear browning identification are discussed. Firstly, the spectra are preprocessed, and the qualitative analysis models of partial least squares discriminant analysis (PLS-DA), the random forest (RF) method, and convolutional neural networks (CNNs) are established. The effects of different spectral preprocessing methods on the prediction accuracy of the above models are discussed. Secondly, the Gramian angular difference field (GADF) is used to convert the spectra of the above two datasets into images, and four models, including PLS-DA, RF, CNNs, and coordinate attention convolutional neural networks (CACNNs), are established based on the images. The advantages of the GADF in constructing spectral spatial domain information are determined. Finally, the noisy spectra are constructed by adding random noise, and various discriminant models representing the three modeling strategies of the SL, CL, and DL methods, respectively, are established to examine the robustness of each model under different noise levels. This research work is expected to fully clarify and utilize the advantages of DL methods in feature capture and simplify the construction process of DL models for NIRS analysis.

## 2. Theory and Algorithm

### 2.1. Gramian Angular Field (GAF)

GAF is a coding method for converting 1D signals to 2D images [27] that can retain the mutual difference or sum information of the original 1D signals. The specific calculation process is as follows.

First, the true value of the spectrum signal $X=\{x_{1},x_{2},\ldots x_{n}\}$ is scaled into the range [0, 1]. The calculation formula is shown in Equation (1).

(1) $${\tilde{x}}_{i}=\frac{(x_{i}-\min(X))}{\max(X)-\min(X)}.$$

where max(*X*) and min(*X*) represent the maximum and minimum values of the spectrum, respectively, and ${\tilde{x}}_{i}$ represents the scaled result of each element in the spectrum.

Second, the arccosine value $\theta_{i}$ of each element of the scaled spectrum $\tilde{X}=\{{\tilde{x}}_{1},{\tilde{x}}_{2},\ldots {\tilde{x}}_{n}\}$ is calculated, and the radius R of the polar coordinates is divided into *N* segments to mark the position information of spectrum t at *N* sampling points. The calculation process is shown in Equation (2).

(2) $$\left[\begin{array}{l} \theta_{i}=\arccos({\tilde{x}}_{i}),\;0\leq {\tilde{x}}_{i}\leq 1,\;{\tilde{x}}_{i}\in \tilde{X}\;\\ r_{i}=\frac{i}{N},\;\;\;\;\;\;\;\;\;\;\;\;\;\;\;with\;\;t_{i}\leq N \end{array}\right].$$

Finally, according to the definition of Equation (3), the value of each pixel in the GAF image is obtained by using the trigonometric function to identify the difference information in different sampling intervals, where I is a unit row vector. A way to preserve the structure dependency is provided, since the spatial position of the image represents the position of the sampling point. The piecewise proximate aggregation (PPA) of the raw data during converting can be seen as a method of spectra smoothing.

(3) $$\begin{array}{l} GADF=[\sin(\theta_{i}-\theta_{j})]={\sqrt{I-{\tilde{X}}^{2}}}^{\prime }\cdot \tilde{X}-{\tilde{X}}^{\prime }\cdot \sqrt{I-{\tilde{X}}^{2}}.\\ GASF=[\cos(\theta_{i}+\theta_{j})]={\tilde{X}}^{\prime }\cdot \tilde{X}-{\sqrt{I-{\tilde{X}}^{2}}}^{\prime }\cdot \sqrt{I-{\tilde{X}}^{2}}. \end{array}$$

### 2.2. PLS-DA

PLS-DA is derived from PLS regression [28] and involves forming a regression model between the X and Y. It assumes that there is a decomposition of ***X*** and ***Y***, as shown in Equation (4).

(4) $$Y=TQ^{T}+F,\;X=TP^{T}+E$$

where $T\;(T\in R^{n\times r})$ is the *r* principal components composed of linear combinations of the observed values; $P\;(P\in R^{p\times r})$ and $Q\;(Q\in R^{1\times r})$ are the coefficient matrix of *r* principal components; and $E\;(E\in R^{n\times p})$ and $F\;(F\in R^{n\times 1})$ are the residual matrix. A set of vectors ***W*** (*W* = *w*_1_, *w*_2_, …, *w_r_*) is found under the condition of Equation (4), and this process follows the optimization standard in Equation (5).

(5) $$\left\{\begin{array}{c} {\hat{w}}_{k}=\arg\max_{w}w^{T}X^{T}YY^{T}Xw\\ s.t.\;w^{T}w=1\;,\;w^{T}S_{XX}{\hat{w}}_{j}=0\;,\;j=0,1,\cdots ,r-1 \end{array}\right.$$

where w is the linear combination coefficient of each row in X and SXX is the sample variance of X. The algorithm described above improves the predictive performance in high-dimensional data by reducing the dimensionality of the independent variables to capture the maximum common variance with the response. The final model can be summarized as Equation (6).

(6) $$\hat{Y}=XWB^{T}+b_{0}$$

where $\hat{Y}$ is the predicted value; ***X*** (*m* × *n*) represents *m* samples, each with *n* features; ***B*** is the transpose of ***P***; and *b*_0_ is the deviation. PLS pays attention to the influence of the extracted components on the dependent variable during the principal component extraction. Therefore, PLS considerably retains the useful information of the data while considering the decisiveness of the independent variable to the dependent variable.

In PLS-DA, ***Y*** is a set of discrete variables representing categories that are usually represented by +1, −1 or +1, 0. However, PLS is designed for continuous variables (regression tasks), and the predicted output is continuous variables. Therefore, Decision Rules (DRs) are used to accurately convert the variables into meaningful category labels. There are three popular DR methods: (a) naive (maximum value, Max); (b) cut-off point; and (c) boundary line [28]. The DR method of the PLS-DA algorithm involved in this article is naive (maximum value, Max).

### 2.3. Random Forest (RF)

The RF method is one of the most widely used CL models, constructing an ensemble model by integrating the results of multiple sub-models developed with different training data subsets formed by using random sampling methods [29]. It consists of multiple decision trees (DTs) generated based on a combination of bootstrap aggregation and the random subspace method. Specifically, bootstrap aggregation is a method to construct multiple data subsets by extracting samples from the original dataset. The random subspace method randomly selects a feature subset as a candidate for splitting when constructing each split node of the decision tree. This further increases the diversity of the models and helps to prevent overfitting. Each decision tree is generated based on CARTs (classification and regression trees). In this study, the Gini index minimization criterion is used for the feature selection, and the binary tree is generated recursively. The calculation formula for the Gini index is shown in Equation (7).

(7) $$\mathrm{Gini}\;\mathrm{indx}(p)=\sum_{k=1}^{K}p_{k}(1-p_{k})$$

where *p_k_* is the frequency of category *k* appearing in the dataset. The DT can select features that are more important for classification tasks by minimizing the Gini index [30].

In the RF, each DT segments the data based on different features and generates a set of rules for predicting the target variables. During the construction of the DTs, randomness is introduced through sampling with replacements from the original data and considering only a subset of features for the node division. Each DT in the RF makes independent predictions during testing, and classification problems are resolved by voting to select the category with the most votes as the final prediction result. Due to differences in the sample subsets selected by each DT and the integration of multiple DTs’ prediction results, the RF is relatively robust against missing data and outliers [31].

### 2.4. Coordinated Attention Convolutional Neural Networks (CACNNs)

A convolutional neural network (CNN) is a variant of a multilayer perceptron (MLP) and uses convolutional operations to capture the features of data. It has been widely used in the classification of images, sound, and other datasets [32]. A CNN is usually composed of three parts: the convolution layer, pooling layer, and fully connected layer. The convolution layer extracts different features from the image through many different convolution kernels, and the features are kept in different channels. Different activation functions (AFs) are used to filter out some of these features. The pooling layer is adapted to retain an important part of the features extracted by the convolutional layer, leading to a reduction in the parameters. The fully connected layers are used to classify the extracted features.

**Figure 1:**
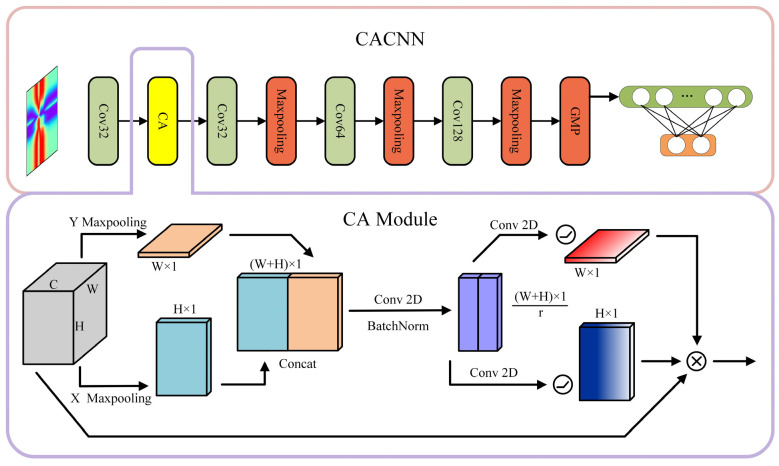
Network structure and CA internal structure. The CNN is composed of a series of convolutional layers and max pooling layers. The fully connected layer is then utilized for the final classification. By incorporating a CA module at the front of the network, the CACNNA network is formed. The CA module embeds positional information by pooling, concatenation, and convolution operations on the initial feature map in two directions.

Many excellent CNNs have emerged in the evolution of network structures. Among these classic networks such as LeNet-5, VGG, GoogLeNet, ResNet, etc., the VGG networks are widely used in image recognition due to their concise structure, small convolutional kernels, small pooling kernels, multiple channels, and feature maps with deeper and wider layers [33]. Each convolution layer uses a 3 × 3 convolution kernel. Based on this network, a coordinate attention (CA) module can help the model better locate and focus on important features. This is added to the CNN to form a CACNN. The network structure is shown in Figure 1.

The general attention module compresses spatial information into the channel descriptor through global pooling, but it is not easy to save the position information. CA comprises two parts: coordinate attention embedding and coordinate attention generation. CA captures and strengthens the spatial details of the essential features in the new feature map. It processes each channel across horizontal and vertical dimensions using a pooling kernel, creating a global perception field that includes the exact position. The above results are concatenated and then transformed using the convolutional transformation function. Another two convolutional transformations change the encoded image into a tensor with the same number of channels as the input.

In this way, long-range dependencies can be captured along one spatial direction, while precise position information can be preserved along another spatial direction. The resulting feature maps are encoded into two types of attention maps: “direction-aware” and “position-aware.” These attention maps complement the input feature maps, focusing on the areas of interest and improving their representation.

## 3. Datasets and Experiments

### 3.1. Datasets

#### 3.1.1. Wheat Kernel Dataset

The wheat kernel datasets contain the reflection spectra of various wheat kernels. The wheat kernels were obtained from a seed company (RunYang, Yangzhou, Jiangsu, China) in Jiangsu Province, China, in 2019 [34]. They were kept under the same environmental conditions after harvest (dried, packaged in woven plastic bags, and delivered to the laboratory). The spectral images of the wheat kernels were collected by an NIR hyperspectral system with a spectral range from 874 to 1734 nm, and the NIRS spectra were extracted from the hyperspectral images. The dataset can be found online at https://www.frontiersin.org/articles/10.3389/fpls.2020.575810 (accessed on 15 August 2024).

The experiment in this article involved 200 samples randomly selected from both huaimai41 (HM41) and jiangmai919 (JM919), totaling 400 samples for the analysis dataset. HM41 and JM919 both belong to the grain-producing area of northern Jiangsu, where the wheat kernels are white, slender, and have a solid texture, making it difficult to classify them by appearance. The variety of wheat kernels is a matter of great concern for growers and consumers because of their different components, resistance, and economic value. The NIRS technology is suitable for this task, due to its characteristics of being rapid and non-destructive. It is difficult to collect a large number of spectral samples in the actual detection tasks, and the random sampling of the original dataset simulates this situation. Therefore, this measure will be beneficial for the application of this research to other spectral datasets. The random sampling method was used to divide the training set and the test set at a ratio of 2:1.

#### 3.1.2. Yali Pear Dataset

**Figure 2:**
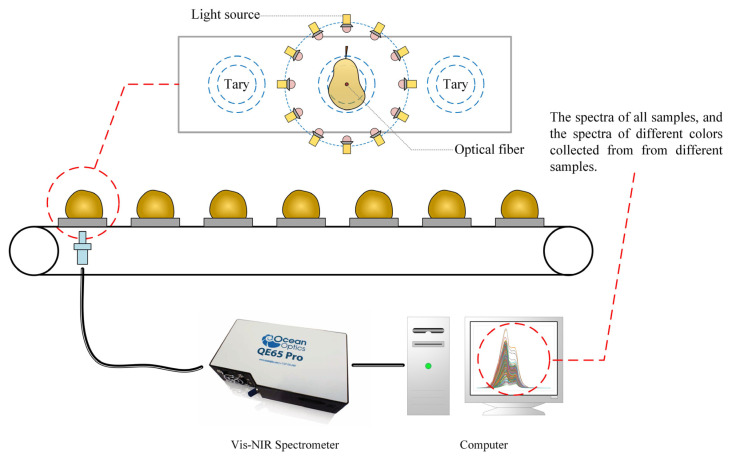
The spectral collection system for Yali pears.

Yali pear browning is a kind of pear heart disease caused by changes in temperature and a long time during storage and transportation. The Yali pear samples were collected from an orchard (LingGuo, Handan, Hebei, China) in Hebei Province, China. After picking, they were transported to the laboratory through cold chain transportation. Before the experiment, these samples were stored at a constant temperature for 24 h, which can reduce the influence of temperature on spectral acquisition [35]. The pears selected for the experiment were about 65–75 mm in diameter, and the skin had no noticeable discoloration or damage. The Vis-NIR spectra of the samples were collected by an Ocean Optics INC, QE-65Pro (Dunedin, FL, USA) high-precision spectrometer. The samples were placed on a tray and sequentially entered the light shield on a conveyor belt for the spectral collection. The spectral acquisition system is shown in Figure 2. The collected signal was the light intensity measured by the sensor after the light source had transmitted through the sample, and the background spectrum was automatically subtracted from the instrument. After the spectra collection, the samples were cut one-by-one along the center line, and three experienced agricultural experts judged whether browning occurred. A total of 495 pear samples were collected, and the spectral collection range was 370–1160 nm, with a total of 1024 data points, including 256 healthy pears and 239 browning pears. The random sampling method was used to divide the training set and the test set at a ratio of 2:1.

### 3.2. Model Evaluation

The performance of the model is evaluated by classification accuracy (Accuracy), positive sample classification accuracy (RP), and negative sample classification accuracy (RN). The closer the values of the above three evaluation indicators are to 100%, the stronger the classification ability of the model. The accuracy is the primary metrics, while the RP and RN are used to test whether the model is biased towards a specific class. The calculation formulas of Accuracy, RP, and RN are shown in Equation (8).

Where *P* is the total number of positive samples; *N* is the total number of negative samples; *P_e_* is the number of positive samples misclassified as negative samples; *N_e_* is the number of negative samples misclassified as positive samples. In the wheat kernel dataset, the HM41 is defined as positive sample, and the is JM919 defined as negative sample. The positive sample in the Yali pear dataset is healthy pear, and the negative sample is browning pear.

(8) $$\begin{array}{c} \mathrm{Accuracy}=(1-\frac{P_{e}+N_{e}}{P+N})\times 100\%\\ \mathrm{RP}=(1-\frac{P_{e}}{P})\times 100\%\\ \mathrm{RN}=(1-\frac{N_{e}}{N})\times 100\% \end{array}$$

### 3.3. Experiments

**Figure 3:**
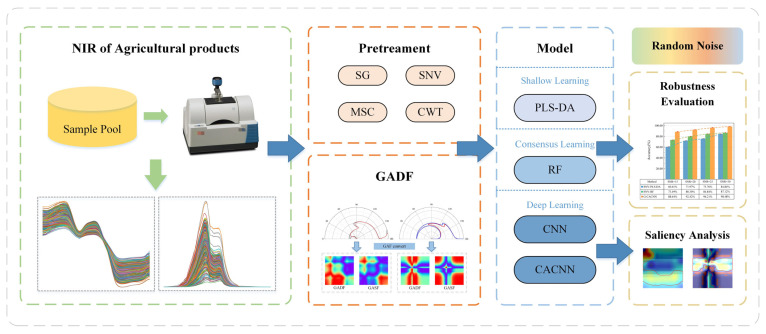
Experimental flow chart. The experiment is mainly composed of four stages. Initially, the spectra for agricultural products is collected. Following that, a variety of processing techniques are utilized to treat the spectra. Subsequently, discriminative models that are representative are developed according to the modeling strategy. Finally, an in-depth analysis of the modeling process is performed.

The experimental process is shown in Figure 3. First, the raw spectra were pretreated with different preprocessing methods, and the classification models, including PLS-DA, RF, and CNN, were developed based on the SL, CL, and DL modeling strategies, respectively. The preprocessing methods involved in the experiment included no preprocessing (None), the Savitzky–Golay filter (SG1st), the standard normal variate (SNV), the multivariate scattering correction (MSC), and the continuous wavelet transform (CWT) methods. The wavelet basis function used by the CWT method was ‘db8’, and the noise threshold was 0.1. To ensure the generalizability and reliability of the modeling analysis, we randomly partitioned and modeled the dataset 10 times and took the mean of the evaluation indicators as the final result. Secondly, the GADF was used to convert the NIRS to images, and four models, including PLS-DA, RF, CNN, and CACNN, were built based on the images and then used again for the modeling. The results of the above four models were analyzed and compared. Finally, noisy datasets were introduced by artificially adding noise, and based on the optimal preprocessing method, the PLS-DA, RF, and CACNN models were established and compared. The robustness of the SL model, CL model, and DL model under different noise influences was discussed. At the same time, the influence of the GADF and CA attention on the models were analyzed by using the saliency visualization method.

The following are the algorithm optimization methods and hyperparameter settings involved in the experiment, including grid search and cross-validation, to ensure the methodology is replicable. For the PLS-DA, Leave-One-Out Cross-Validation (LOOCV) was employed as the cross-validation strategy to assess the model performance, ensuring that each sample had the opportunity to serve as a test set, thereby enhancing the reliability of the model evaluation. The optimal number of components was within the range of 1 to 15.

For the RF, the grid search method was applied to optimize the number of decision trees and the maximum depth of the decision trees, two critical hyperparameters in the model. The search interval for the number of decision trees ranged from 50 to 300 with a step size of 50, covering a wide range of model complexities. At the maximum depth, the search interval was from 10 to 100 with a step size of 20 to prevent overfitting and underfitting.

In the training of the DL models, the initial learning rate was 0.0005, which decayed by 50% every 200 iterations, for a total of 600 iterations. To prevent overfitting, a dropout optimization method was added after the fully connected layer, with a dropout rate set to 0.25. The structure and parameter settings of the CNN are shown in Table 1. The structure of the CACNN network was roughly the same as that in Table 1, with the CA attention layer inserted between Conv1 and Conv2.

All data processing and modeling experiments were performed on a personal computer (CPU: Intel i5-12400f (Intel, Santa Clara, CA, USA); and GPU: GeForce RTX 2060 Super (Nvidia, Santa Clara, CA, USA)). The software environment was Python3.7.0, and the DL model was built using the TensorFlow architecture.

## 4. Results and Analysis

### 4.1. Spectral Analysis and GAF Converting

**Figure 4:**
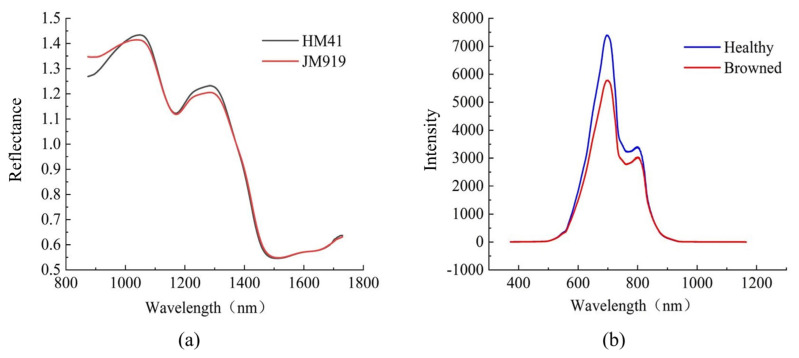
Average spectra. (**a**) The average spectra of wheat kernels. (**b**) The average spectra of Yali pears.

The average spectra of the two categories of wheat kernels are shown in Figure 4a. The difference between the average spectra of the two categories of samples in the 870–1100 nm and 1200–1400 nm bands is significant. The absorbance peaks of different bands in the following wavelengths are most noteworthy: the 965–985 nm N-H stretching second overtone band associated with proteins; the 1130–1200 nm second overtone of C-H stretching, which is related to carbohydrates; 1330–1385 nm, a combination of C-H stretching and C-H deformation [36].

The average spectra of the two categories of Yali pears are shown in Figure 4b. The average spectrum of healthy pears is higher than that of browned pears on the whole, and the band of 600–800 nm is the most obvious, which may be due to the strong absorption of transmitted light by the browning tissue inside the fruit. As the spectra show, there are two absorption peaks at approximately 700 and 800 nm. The absorption peak at around 700 nm may result from the stretching and contraction of the fourth overtone of the C-H functional group, while that at around 800 nm may be related to the stretching and contraction of the third overtone of the N-H functional group [37].

**Figure 5:**
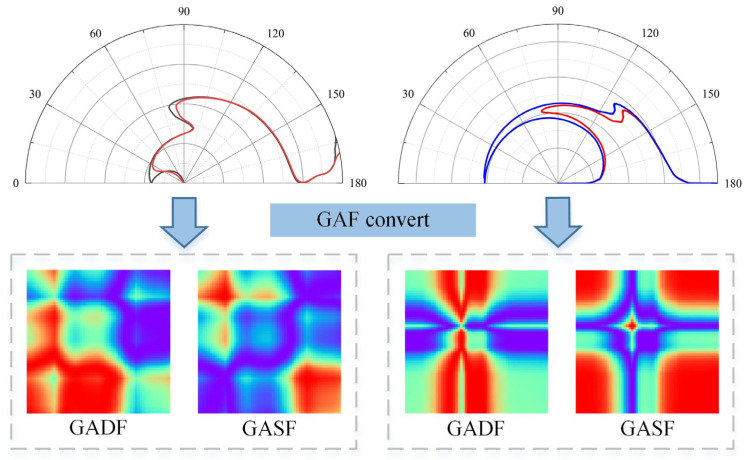
Schematic diagram of GAF converting process. The color change from blue to red corresponds to the increment of the value in the pixel.

The GAF encoding process of the average spectra of the two datasets is shown in Figure 5. After the polar coordinate transformation, the NIR numerical fluctuations are transformed into angular changes. The encoded GASF image is symmetrical along the main diagonal, and the main diagonal represents the original 1D NIRS sequence. In the GADF image, the main diagonal is always 0, and the pixel values of the two symmetrical points along the main diagonal are opposite to each other, which is caused by the exchange of the difference order. The spectral feature information is enriched with GADF by exploring the difference in the value of different positions in a single spectral sequence.

### 4.2. Spectra Discriminative Model Analysis

#### 4.2.1. Wheat Kernel Dataset

The results of the PLS-DA, RF, and CNN discrimination models for wheat kernels are shown in Table 2, and the influence of different spectral preprocessing methods on the modeling results of the above various models is analyzed. The standard deviation (SD) between each set of evaluation indicators is used to measure the impact of the preprocessing methods on the modeling results. The smaller the standard deviation of accuracy across the different preprocessing methods, the more stable the modeling method. From the table, it can be seen that different preprocessing methods have the greatest influence on the PLS-DA model, and the SD of the accuracy is 1.24%. The average accuracies of the three modeling methods are 96.68%, 96.69%, and 97.18%, respectively. The SD of the training accuracy of several preprocessing methods for the RF model is 0.62%. The CNN model is less affected by different preprocessing methods, and the SD of the accuracy is only 0.53%. For the PLS-DA model, the optimal model result of the spectra pretreated by SNV is 97.78%. The analysis results of the RF and CNN models varied slightly with the different spectral preprocessing methods and had good stability. Moreover, the CNN model of the original spectra could obtain similar analysis results to the optimal SNV-PLS-DA model. The spectral preprocessing methods had little influence on the analysis results of the DL model, and the model showed better robustness. The results of the deep model on the original spectra were still good.

#### 4.2.2. Yali Pear Dataset

The results of the PLS-DA, RF, and CNN discrimination models for Yali pears are shown in Table 3. Similar to the above analysis, the spectral preprocessing methods have different effects on the model analysis results. Among them, the preprocessing methods have the greatest influence on the analysis results of the PLS-DA model, and the SD of the accuracy of the modeling results with different preprocessing methods is 4.72%. The RF model and the CNN model are less affected, and the SD of the accuracy of varying preprocessing methods are 0.65% and 0.48%, respectively. The average accuracies of the three modeling methods are 91.49%, 87.44%, and 95.67%, respectively. For the task of identifying Yali pear browning, the accuracy of the CNN modeling is significantly better than the other two models. Moreover, the result of the CNN without any spectral preprocessing methods is much higher than that of the other two models, which is close to the optimal result of the PLS-DA of 95.41%. The results show that the DL model can complete the qualitative analysis of samples based on NIRS without complex spectral preprocessing.

### 4.3. Advantages of Images in Modeling

For the images, the CNN and CACNN were used for the modeling and prediction, respectively. A 64 × 64 pixel image was flattened into a one-dimensional vector of length 4096 according to row-major order rules. For the vectors, the RF and PLS-DA were used for modeling and prediction, respectively. Table 4 shows the results of all models developed with GADF images. Compared with the results with the spectra, the accuracy of both the PLS-DA and CNN were improved, which shows that the GADF can effectively enhance the spectral feature information. For several modeling methods, the ability of the CNN to process images was superior to that of the traditional PLS-DA and RF models, and the CA module could further improve the modeling results of the CNN. The method proposed in this paper has achieved the best results in a series of experiments: the accuracy of the wheat kernel classification is 98.48% and the accuracy of the Yali pear classification is 99.39%.

### 4.4. Optimal Model Analysis

The convolution kernels are used to scan images and extract different features, which have a receptive field. The regional scanning makes the different variables (pixels) no longer independent, which means the different arrangements of variables are bound to affect the modeling results. When the spectra are convoluted and scanned, only small-span spectral information can be displayed in the local area. When the convolution operation is performed on the image encoded by the GADF, the small span can reflect long-range spectral information. A CNN is not sensitive to the global position of features, and it is generally agreed that it is biased toward whether there are decisive features in a small range. From this point of view, the dataset encoded by the GADF is more suitable for the CNN feature capture preferences.

**Figure 6:**
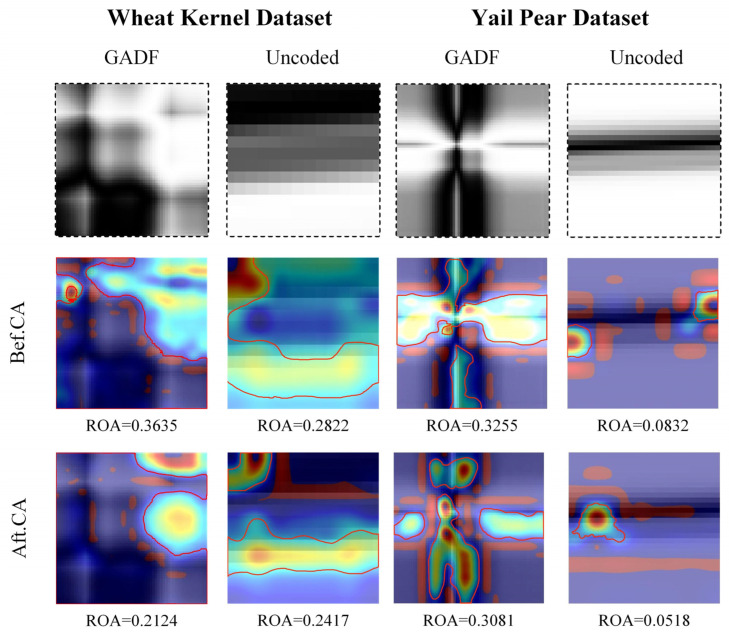
Grad-CAM of DL model. The yellow area is paid attention to by the network, and the blue area network lacks attention. The red line is the dividing line of the attention heatmap. The attention concentration of the model is evaluated by dividing the heatmap into regions of interest and regions of non-interest.

Gradient-weighted Class Activation Mapping (Grad-CAM) is used to visualize the attention distribution of the network. The Grad-CAM is generated based on the unique contributions of each feature map channel. It integrates the prediction outcome with the gradients across the feature maps, thereby highlighting the critical areas that influence the network’s classification. The first line of Figure 6 shows the grayscale image input into the network, the grayscale image facilitates coverage of the attention heat map. The second line shows the Grad-CAM of the four methods of CNNs, CACNNs, G-CNNs, and G-CACNNs before adding the CA module, and the second line shows the Grad-CAM of the four methods after adding the CA module. The yellow area is paid attention to by the network, and the blue area network lacks attention. The ratio of attention (ROA) refers to the ratio of the area in the entire image where the attention weight exceeds a certain threshold. In this paper, the ROA is used to evaluate the degree of network attention concentration.

The CNN perceives particular features of the uncoded spectrum through a complex mechanism and makes relatively correct judgments but cannot reasonably explain them. However, the attention area of the network on the GADF spectral image is interpretable. Taking the spectra of different samples in the Yali pear dataset as an example, there are differences in the shape of the NIR absorption peaks between different types of samples. These differences are encoded by the GADF and fall into specific areas of the image. In particular, the network attention area coincides with the different information areas. The CNN can effectively extract different characteristics of the absorption peaks from different samples and make a classification, which has been proven. Compared with the DL model of unencoded spectral images, the model trained with the GADF spectral images is more interpretable and has a higher accuracy (Section 4.3).

For the wheat kernel dataset, the ROA of the G-CNN network decreased from 0.3635 to 0.2124 after adding the CA module. For the Yali pear dataset, the ROA of the G-GACNN network decreased from 0.3255 to 0.3081 after adding the CA module. The G-CACNN pays more attention to the information regions.

### 4.5. Robustness Analysis of the Models

**Figure 7:**
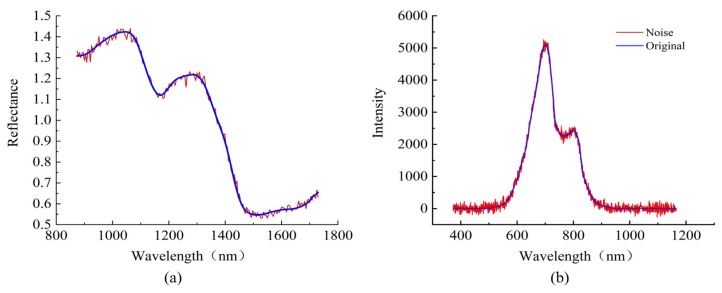
Schematic diagram of additive noise of spectra. (**a**) Wheat kernel dataset. (**b**) Yali pear dataset.

To assess the robustness of the models, Gaussian white noise was deliberately introduced to the original spectra. Gaussian white noise, commonly found in electronic measurements and imaging systems, was selected to simulate the inherent variability and uncertainties that arise from instrumental imperfections during spectral acquisition. The signal-to-noise ratio (SNR) served as the metric for gauging the noise level within the spectra. A higher SNR indicates a clearer signal, while a lower SNR represents a more noisy environment, which can challenge the model’s predictive capabilities. The SNR calculation, as referenced in Equation (9), is pivotal in this evaluation process. It provides a quantitative measure of the noise level relative to the signal strength, allowing for a systematic approach to model assessment across different noise conditions.

(9) $$\mathrm{SNR}\;=10\;lg\frac{P_{s}}{P_{n}}$$

where *P_s_* is the power of the useful signal, *P_n_* is the power of the noise signal, and lg represents the logarithm based on 10. This work simulates spectra data acquisition with additive noise under harsh acquisition conditions. Figure 7 shows the average spectra before and after adding noise.

**Figure 8:**
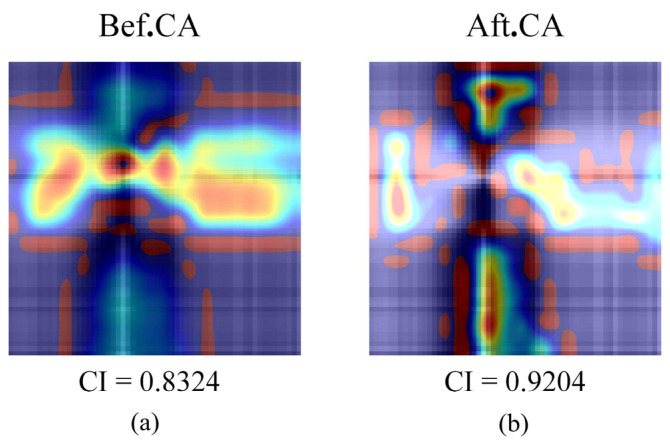
Grad-CAM results with noise spectrum. The yellow area is paid attention to by the network, and the blue area network lacks attention. (**a**) G-CNN. (**b**) G-CACNN.

The confidence index (CI) is the basis on which networks are ultimately classified. The higher the CI, the greater the likelihood that the unknown sample belongs to the class. In the CACNN model, the CI is represented by the outputs of the final fully connected layer, which are transformed into a probability distribution after processing by the softmax function. Figure 8 illustrates the Grad-CAM and confidence index of the seventh healthy pear noise (SNR = 20) spectral encoding map modeled with the G-CNN and G-CACNN. Before using the CA module, the CI was 0.8324. The attention of the network was focused on a few points after adding the CA module and the CI increased to 0.9204. In image classification, the CA module encodes positional information and fuses it with feature variables. Through iterative training, the model will assign more significant weights to the feature variable locations. Consequently, this reduces the impact of non-essential areas on the identification results, thus improving the model’s accuracy.

**Figure 9:**
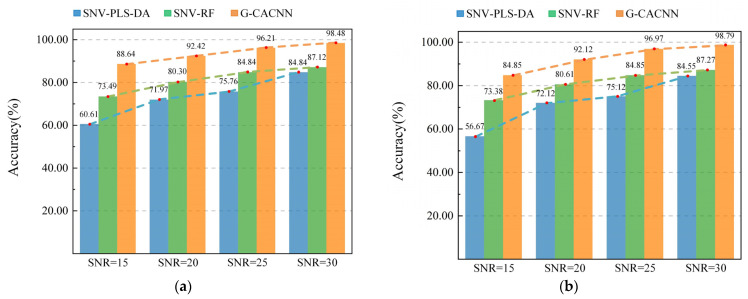
Results of different methods under different levels of noise. (**a**) Robustness test of the models based on the wheat kernel dataset. (**b**) Robustness test of the models based on the pear dataset.

The introduction of the CA module makes the CNN achieve a better performance. At the same time, the noise immunity of the model is also enhanced. The models were constructed using PLS-DA and the RF after the SNV of the noise spectra in the presence of additive noise. Figure 9 shows the comparison of the modeling influence of the proposed preprocessed SL model, CL model, and DL model under different levels of noise.

As Figure 9 shows, with the improvement of the SNR, the prediction accuracies of the three types of models have different improvements. The PLS-DA is very sensitive to noise in the data, so the preprocessing method cannot eliminate the influence of spectral noise on its analysis results. Under the same level of noise conditions, PLS-DA always has the lowest prediction accuracy among the three models. In contrast, the EL model SNV-RF performs better, which demonstrates its robustness to noisy data. The result of the GADF-CACNN is better than the SNV-RF at all noise levels. For the wheat kernel dataset, the results are 88.64%, 92.42%, 96.21%, and 98.48%. For the pear dataset, they are 84.85%, 92.12%, 96.97%, and 98.79%. This means the method has good noise immunity and the best robustness when dealing with spectral data with additive noise. At the same time, from the perspective of the changing trend of accuracy, the effect of the change of the noise level on the DL model and the ensemble model is less than on the SL model, and the influence of the GADF-CACNN is the least, which is also an important feature to demonstrate the robust operation of the model.

## 5. Conclusions

In this paper, a robust NIRS modeling method based on the GADF and CACNN methods is proposed. The GADF method converts 1D spectral signals into 2D images, enriching and highlighting the spectral characteristics. The CACNN network is designed to make full use of the excellent feature extraction ability of the DL model, and the CA module is added to improve the prediction accuracy of the deep structure model. Without using preprocessing, the GADF-CACNN model has an accuracy of 99.39% in the pear browning classification and an accuracy of 98.48% in the wheat kernel classification. In addition, the robustness analysis of the traditional shallow structure model and the GADF-CACNN model is carried out, and the experimental results show that the result of the DL method is better than those of the SL model and the CL model. The method proposed in this paper has the characteristics of simple modeling steps and good model interpretability with good accuracy and robustness for the NIRS analysis.

## Figures and Tables

**Table 1:** The structure and parameters of the CNN.

Layers	Size	Number	Activation	Output
Input	64 × 64 × 1	-	-	-
Conv1	3 × 3	32	ReLU	64 × 64 × 32
Conv2	3 × 3	32	ReLU	64 × 64 × 32
Max-Pooling1	2 × 2	-	-	32 × 32 × 32
Conv3	3 × 3	64	ReLU	32 × 32 × 64
Max-Pooling2	2 × 2	-	-	16 × 16 × 64
Conv4	3 × 3	128	ReLU	16 × 16 × 128
Max-Pooling3	2 × 2	-	-	8 × 8 × 128
GlobalMaxPooling2D	-	-		128
Dense1	128	-	ReLU	128
Dense2	2	-	Softmax	2

**Table 2:** Wheat kernel dataset modeling results.

Classifier	Pretreatment	Accuracy (%)	RP (%)	RN (%)
PLS-DA	None	94.73	96.75	92.34
	SG-1st	96.35	96.64	95.63
	**SNV**	**97.78**	**98.53**	**97.11**
	MSC	97.69	98.41	97.13
	CWT	96.83	96.73	97.16
	-	96.68 ± 1.24 ^a^	97.41 ± 0.97	95.87 ± 2.08
RF	None	95.97	96.61	95.5
	SG-1st	96.47	98.31	95.56
	SNV	97.61	98.44	97.06
	**MSC**	**96.93**	**96.71**	**97.13**
	CWT	96.47	96.81	95.62
	-	96.69 ± 0.62	97.38 ± 0.92	96.17 ± 0.84
CNN	None	96.86	98.42	95.54
	SG-1st	96.93	98.41	95.56
	SNV	97.73	98.44	97.06
	**MSC**	**97.81**	**98.44**	**97.06**
	CWT	96.63	96.71	96.46
	-	97.18 ± 0.53	98.08 ± 0.77	92.82 ± 0.76

^a^: mean ± SD of evaluation indicators.

**Table 3:** Yali pear dataset modeling results.

Classifier	Pretreatment	Accuracy (%)	RP (%)	RN (%)
PLS-DA	None	84.2	80.16	87.69
	SG-1st	89.47	90.71	88.96
	**SNV**	**95.41**	**94.57**	**96.61**
	MSC	95.24	94.18	98.13
	CWT	93.11	93.48	92.73
	-	91.49 ± 4.72 ^a^	90.62 ± 6.04	92.82 ± 4.58
RF	None	86.64	86.02	86.79
	SG-1st	87.22	88.41	86.49
	**SNV**	**88.41**	**88.13**	**88.74**
	MSC	87.31	86.82	87.69
	CWT	87.62	88.91	87.24
	-	87.44 ± 0.65	87.66 ± 1.02	87.39 ± 0.88
CNN	None	95.37	95.16	95.04
	SG-1st	95.86	94.81	96.6
	**SNV**	**96.41**	**96.73**	**96.11**
	MSC	95.18	95.47	96.63
	CWT	95.54	94.49	96.51
	-	95.67 ± 0.48	95.33 ± 0.86	96.18 ± 0.67

^a^: mean ± SD of evaluation indicators.

**Table 4:** Modeling results based on GADF images.

Model	Wheat Kernel Dataset			Yali Pear Dataset		
	Accuracy (%)	RP (%)	RN (%)	Accuracy (%)	RP (%)	RN (%)
G-PLS-DA	95.45	96.88	94.12	90.91	92.31	88.24
G-RF	94.96	95.31	95.45	96.88	94.12	88.52
G-CNN	96.97	97.06	96.35	97.98	98.46	97.06
**G-CACNN**	**98.48**	**98.44**	**98.53**	**99.39**	**100**	**98.36**

## Data Availability

The data presented in this study are available on request from the corresponding author, due to the data needing to be used for subsequent research or further analysis.
